# HFS-SLPEE: A Novel Hierarchical Feature Selection and Second Learning Probability Error Ensemble Model for Precision Cancer Diagnosis

**DOI:** 10.3389/fcell.2021.696359

**Published:** 2021-06-30

**Authors:** Yajie Meng, Min Jin

**Affiliations:** College of Computer Science and Electronic Engineering, Hunan University, Changsha, China

**Keywords:** precision cancer diagnosis, hierarchical feature selection, ensemble model, transcriptome profiling, DNA methylation, biomarker

## Abstract

The emergence of high-throughput RNA-seq data has offered unprecedented opportunities for cancer diagnosis. However, capturing biological data with highly nonlinear and complex associations by most existing approaches for cancer diagnosis has been challenging. In this study, we propose a novel hierarchical feature selection and second learning probability error ensemble model (named HFS-SLPEE) for precision cancer diagnosis. Specifically, we first integrated protein-coding gene expression profiles, non-coding RNA expression profiles, and DNA methylation data to provide rich information; afterward, we designed a novel hierarchical feature selection method, which takes the CpG-gene biological associations into account and can select a compact set of superior features; next, we used four individual classifiers with significant differences and apparent complementary to build the heterogeneous classifiers; lastly, we developed a second learning probability error ensemble model called SLPEE to thoroughly learn the new data consisting of classifiers-predicted class probability values and the actual label, further realizing the self-correction of the diagnosis errors. Benchmarking comparisons on TCGA showed that HFS-SLPEE performs better than the state-of-the-art approaches. Moreover, we analyzed in-depth 10 groups of selected features and found several novel HFS-SLPEE-predicted epigenomics and epigenetics biomarkers for breast invasive carcinoma (BRCA) (e.g., TSLP and ADAMTS9-AS2), lung adenocarcinoma (LUAD) (e.g., HBA1 and CTB-43E15.1), and kidney renal clear cell carcinoma (KIRC) (e.g., IRX2 and BMPR1B-AS1).

## Introduction

Cancer has the characteristics of concealed onset, low cure rate, and high mortality. Traditional surgery, radiotherapy, and chemotherapy have limited effects on patients with advanced cancer. The cancer diagnosis is currently a hot research topic. With the acquisition of numerous gene expression profiles in various tissue samples, it is possible to perform cancer diagnosis at the molecular level. Early biology supposed that cancer is closely related to the mutations of protein-coding genes (Stratton et al., 2009). Thus, mass studies confirmed the feasibility of cancer diagnosis based on messenger RNA (mRNA) expression profiles, achieving some good results (Ben-Dor et al., 2000; Furey et al., 2000; Li et al., 2001). Recently, biological studies have found that without changing the sequence of the protein-coding genome, there are a lot of epigenetic variations involving multiaspects such as non-coding RNA (ncRNA) and DNA methylation in cancer (Baylin and Ohm, 2006; Luo et al., 2016; Xiao et al., 2018a). Numerous studies utilized epigenetic data such as microRNA (miRNA) (Saha et al., 2015), long non-coding RNA (lncRNA) expression profiles (Zhang et al., 2018), and DNA methylation (Al-Juniad et al., 2018) for cancer diagnosis and subtype classification, obtaining some achievements (Raweh et al., 2018; Tang et al., 2018). However, the latest biological research indicates that multibiomarkers can improve the accuracy and robustness of cancer diagnosis (Modelska et al., 2015). Zhao et al. (2018) integrated protein-coding gene, miRNA, and lncRNA expression profiles for lung adenocarcinoma (LUAD) diagnosis. Alghunaim and Al-Baity (2019) fused protein-coding gene expression profiles and DNA methylation for breast invasive carcinoma (BRCA) diagnosis. Classical genetics and epigenetics are two separate mechanisms participating in carcinogenesis (Network, 2012). Additionally, epigenetics data such as ncRNA and DNA methylation are not independent of each other, and they often have synergistic effects (Xu et al., 2018). Therefore, only using protein-coding gene expression profiles and/or ncRNA expression profiles or DNA methylation data leads to the lack of information and prevents the high-performance and robustness of cancer diagnosis from being significantly improved.

The emergence of large-scale RNA-seq data and DNA methylation data has offered unprecedented opportunities for developing cancer diagnosis approaches. However, integrating transcriptome profiling (i.e., protein-coding gene and ncRNA expression profiles) and DNA methylation data for cancer diagnosis faces challenges. Model et al. (2001); Ang et al. (2015), Gao et al. (2017); Lu et al. (2017), and Sun et al. (2019) pointed out that transcriptome profiling and DNA methylation data are featured with high dimensionality, high redundancy, and complex interaction associations. To solve the problem of high dimensionality, feature scoring functions such as differences and distances between normal and tumor samples, correlation coefficients, and information metrics between features and categories are commonly applied for filtering feature (Lazar et al., 2012). For example, Yoon and Lim (2013) used *t*-test and Euclidean distance, and Cao et al. (2015) adopted the fold-change (FC) and false discovery rate (FDR) for filtering feature. These filter methods efficiently remove irrelevant features to reduce dimensions, which has the characteristics of strong universality and low complexity and are suitable for processing high-dimension data. However, these filter methods are from the perspective of a single feature, without considering the high redundancy between features. Subsequently, Peng et al. (2005) proposed the minimal redundancy and maximal relevance criterion, named mRMR, which based on the maximum correlation between features and categories and the minimum redundancy between features in feature subsets. Lyu et al. (2017) designed LLRFCscore+ algorithm, which first sorts features in descending order *via* LLRFC criteria and then use the dynamic correlation analysis strategy to eliminate redundant features further. Raweh et al. (2018) developed a hybrid feature selection algorithm with a filtering method and a new feature extraction algorithm. Based on informatics theory, these methods can effectively remove redundancy for single-type data. However, these methods calculated the many-to-many modification associations between DNA methylation CpG sites and genes as redundant correlations. Thus, feature selection methods for transcriptome profiling and DNA methylation data are necessary to study further.

The diagnosis models are also essential for cancer diagnosis. Due to the diversity of classifiers, ensemble models tended to have better performance than single models (Dietterich, 2000a; Yang et al., 2010; Zhou and Jin, 2017). Generally speaking, there are three common types of ensemble strategies in the cancer diagnosis field, namely, the voting method, average method, and learning method. For example, Huang et al. (2017) proposed an support vector machine (SVM) ensemble model, which constructed SVM base classifiers with different kernel functions based on bagging and boosting sampling and used majority voting and weighted average ensemble strategies. The SVM ensemble model solves the problems of easy fitting and limited generalization of a single model, but it is limited to the same type classifiers and cannot fully guarantee the difference between classifiers. Cho and Won (2003) trained four different types of individual classifiers and obtained the final ensemble model by majority voting method. This method takes advantage of the complementarity among the different individual classifiers and breaks through the limitation of the application scope of single classifiers. Although it is relatively simple to integrate homogeneous and heterogeneous classifiers by the voting and average methods, it cannot ensemble the nonlinear relationship between classifiers. To further ameliorate the voting and average methods, Xiao et al. (2018b) utilized the stacking learning ensemble strategy, which based on cross-validation to train five different classifiers and put the training results of the classifiers as the input of a deep learning algorithm. The learning ensemble strategy effectively integrate the nonlinear relationships between the heterogeneous classifiers. However, the deep learning algorithm highly depends on the size of samples, whose performance needs to be enhanced by a large increase in the samples of data. Therefore, these ensemble methods show a limited performance for precise cancer diagnosis.

To address the above limitations, we proposed a novel hierarchical feature selection and second learning probability error ensemble model, called HFS-SLPEE, for precision cancer diagnosis. At the dataset level, we integrated protein-coding genes expression profiles, ncRNAs expression profiles, and DNA methylation data to construct a triple dataset that provides a multiview perspective and diverse information. At the feature selection level, due to the significant differences in dimensions, abundance, and association relationships of the triple dataset, we designed a novel hierarchical feature selection algorithm. In stage 1, we developed a CpG sites aggregation feature selection algorithm, termed CSAFS, to non-destructively store the biological associations between ultra-high dimensions DNA methylation CpG sites and genes and rapidly reduce the nearly 500,000 dimensions DNA methylation data to nearly 30,000 dimensions methylated gene data. In stage 2 feature selection, we used different thresholds to further select significantly differentially features *via* FC and FDR. In stage 3 feature selection, we adopted the mRMR algorithm to select a compact set of superior features. At the model diagnosis level, we developed a second learning probability error ensemble model, named SLPEE. Specifically, we selected four individual classifiers with significant differences and apparent complementary effects to build heterogeneous classifiers and then obtain the classifiers-predicted class probability predictions. To thoroughly learn the nonlinear data, SLPEE integrated the classifiers-predicted class probability predictions and the actual class label in the validating set to construct a new training set, which implicitly included the probability error of each classifier. Furthermore, we utilized eXtreme Gradient Boosting (Xgboost) as ensemble learner to secondly learn the new training set. *Via* training on three cancers in The Cancer Genome Atlas (TCGA) based on 10-fold cross-validation, HFS-SLPEE achieved 100% multi-indicators of LUAD and kidney renal clear cell carcinoma (KIRC), and obtained 99.65% accuracy, 99.61% sensitivity, 100% specificity, and 99.81% F1-score of BRCA, outperforming previously published approaches. The results indicate that HFS-SLPEE is an accurate and robust approach for cancer diagnosis.

Theoretically, the three contributions of this work are as follows: (i) we integrate protein-coding gene expression profiles, ncRNA expression profiles, and DNA methylation data to solve the problem of lacking information on cancer diagnosis; (ii) we take the biological complex associations into account and develop a novel hierarchical feature selection approach, which integrates the proposed CSAFS, FC&FDR, mRMR, to efficiently select a group of superior and compact features; and (iii) we design an SLPEE model, which makes predictions by secondly learning the error rules between the predicted class probability values of the heterogeneous classifiers and the real values to realize the self-correction of the diagnosis errors *via* the nonlinear ensemble of heterogeneous classifiers.

## Materials and Methods

**FIGURE 1:**
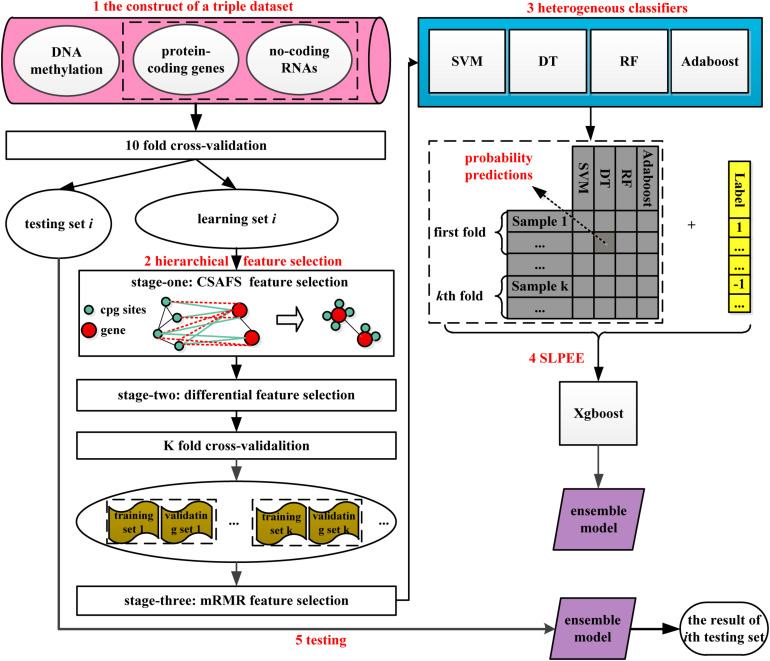
A flowchart of HFS-SLPEE. We first integrate the protein-coding gene expression profiles, non-coding RNA expression profiles, and DNA methylation data to get rich information. Afterward, considering the CpG-gene biological associations, we design a novel hierarchical feature selection method to get a compact group of superior features. Next, we train four heterogeneous classifiers in the training set with the selected features and optimize the parameters of heterogeneous classifiers *via* the grid search algorithm in the validating set. Finally, we develop a second learning probability error ensemble model (named SLPEE) to ensemble the class probability predictions of the heterogeneous classifiers under the optimal parameters. SLPEE is utilized to predict the testing set in each fold. HFS-SLPEE is a precision cancer diagnosis framework, which is powerful tool for precision cancer diagnosis.

As shown in Figure 1, HFS-SLPEE consists of four parts: (i) the construction of a triple dataset, (ii) the novel hierarchical feature selection method, (iii) heterogeneous classifiers, and (iv) SLPEE. First, we integrated three biological data including protein-coding gene expression profiles, ncRNA expression profiles, and DNA methylation data to construct a triple dataset, which contains the rich information. Afterward, we hierarchically selected features for the triple dataset. In the first-stage feature selection, we designed the CSAFS algorithm, which could quickly reduce nearly 500,000 dimensions of DNA methylation data to tens of thousands of dimensions and non-destructively preserved the biological complex modification associations between DNA methylation CpG sites and genes. In the second-stage feature selection, we adopted the FC and FDR to select the features with the highest relevance to the target class. In the third-stage feature selection, we applied the mRMR algorithm to select a compact set of superior features with minimal redundancy and maximal relevance. Next, we trained four heterogeneous classifiers in the training set with the features selected *via* (ii) and optimize the parameters of heterogeneous classifiers *via* the grid search algorithm in the validating set. Finally, we developed the SLPEE model to ensemble the class probability predictions of the heterogeneous classifiers under the optimal parameters. SLPEE was utilized to predict the testing set in each fold and obtained the novel informative biomarkers.

### Construction of the Triple Dataset

The central dogma of classical genetics indicated that genetic information was stored in protein-coding genes (Crick et al., 1961). It had been thought that the formation of tumors is due to the mutations of protein-coding genes. In recent years, plenty of evidence showed that epigenetics also played an essential role in tumor progression (Xu et al., 2019, 2020; Meng et al., 2020). Epigenetics did not involve the DNA sequence changes but changed the structure of chromosomes *via* different mechanisms that affected the activity of surrounding genes to induce cancer. These known mechanisms commonly included the regulation of ncRNA, DNA methylation, and histone modification, but there are many mechanisms that are unknown so far. Herein, we integrated protein-coding gene expression profiles, ncRNA expression profiles, and DNA methylation data to construct a triple dataset, which contained rich information and provided a multiview perspective for precision cancer diagnosis.

### The Novel Hierarchical Feature Selection

The triple dataset exhibits complex characteristics: (i) There were many-to-many complex modification associations between methylation CpG sites and genes; (ii) DNA methylation data were nearly 500,000 dimensions, while the transcriptome profiling were nearly 60,000 dimensions; (iii) there were many noise and redundancy features, and only a small part of the features was positively related to the two phenotypes as tumor and normal tissues; and (iv) the expression abundance of the transcriptome profiling was significantly different from the DNA methylation data. We proposed a novel hierarchical feature selection algorithm against the complex characteristics of the triple dataset.

#### Stage 1: CpG Sites Aggregation Feature Selection

In this study, we used Illumina Human Methylation 450 array methylation chip data, which measures the level of methylation at known CpG sites as follows:

(1) $$\beta =\frac{M}{M+U}$$

where *M* denotes the methylated array intensity, *M+U* denotes the unmethylated array intensity, and β represents the ratio between the methylated array intensity and the total array intensity, ranging from 0 to 1. A CpG site could modify multigenes, while a gene might be related to multi CpG sites. To rapidly reduce the dimensions of the ultra-high dimensionality DNA methylation data and preserve the many-to-many complex biological associations between CpG sites and genes in advance, we proposed a novel CpG sites aggregation feature selection method, called CSAFS, as the stage 1 feature selection algorithm to obtain methylated genes. Specifically, we defined the methylated genes as *MG_gj_*, which represents the arithmetic mean value of the methylation level of all CpG sites related to the gene *g_j_*. The *MG_gj_* is calculated as follows:

(2) $$MG_{g_{j}}=\frac{\sum_{i=1}^{P}CpG_{i}}{P}$$

where *P* denotes the dimensions of the DNA methylation data, *P* = 485,577, *CpG*_*i*_(*i* = 1, 2…,*P*) represents the level of methylation at the known *i*th CpG site, and $\sum_{i=1}^{P}C\,p\,G_{i}$ denotes the aggregate value of all relevant CpG sites of the gene *g_j_*.

#### Stage 2: FC and FDR

The dimensions of methylated genes data and protein-coding gene and ncRNA expression profiles belonged to tens of thousands of dimensions after the stage 1 feature selection. However, the dimensions were still relatively high. Therefore, we utilized FC and FDR statistical methods to further perform overall rapid dimension reduction.

##### Fold Change

FC is a well-known method to screen the differentially expressed genes of microarray data (DeRisi et al., 1996; Schena et al., 1996), which measures the difference through calculating the ratio of the mean value of two groups data (DeRisi et al., 1997). The FC value of the *g*th gene is calculated as follows:

(3) $$FC_{g}=\frac{\frac{\sum_{t=1}^{T}x_{g}^{t}}{T}}{\frac{\sum_{n=1}^{N}y_{g}^{n}}{N}}$$

where *T* is the size of tumor samples, *N* is the size of normal samples, $x_{g}^{t}$ is the *g*th gene expression value of the *t*th tumor sample, $y_{g}^{n}$ is the *g*th gene expression value of the *n*th normal sample. Similarly, the FC values of methylated genes are obtained by calculating the ratio of the mean methylation level of methylated genes in normal and tumor samples. In this study, the thresholds for screening significantly differentially methylated genes and expressed genes are, respectively, (|*log*_2_⁡*FC*| > 0.5) and (*FDR* <  0.05) and (|*log*_2_⁡*FC*| > 3) and (*FDR* <  0.05). Although the differential features selected by FC method have strong repeatability, the false-positive results rate is relatively high in the absence of false-positive control (McCarthy and Smyth, 2009).

##### False Discovery Rate

Without any control, the probability of making the type I error will increase rapidly with the number of hypothesis tests. The FDR could test as many features as possible and effectively control the overall false-positive rate within an acceptable range (Norris and Kahn, 2006). In this study, we applied the Benjamini–Hochberg method to perform multihypothesis test FDR correction on the significant p-value. We adopted *FDR* < 0.05 as the threshold of screening the significant difference feature.

**FIGURE 2:**
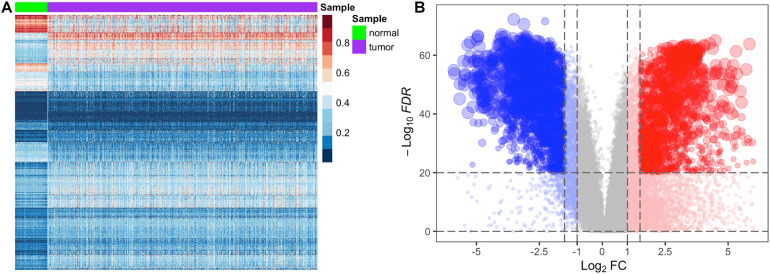
Heatmap analysis of differentially methylated genes and volcano plot analysis of differentially expression genes for the BRCA dataset. In panel **(A)**, the row represents the methylation level of the genes, and the column represents the normal and tumor samples. Dark red shades indicate the higher level of methylation, and dark blue shades indicate the lower level of methylation. Color keys indicate the intensity associated with normalized beta values. In panel **(B)**, the x-axis represents the *log*_2_*FC*, and the y-axis represents −*log*_10_(*FDR*), and each dot represents a gene. The significantly upregulated genes are highlighted in red, and the significantly downregulated genes in blue.

In the stage 3 feature selection, we used FC and FDR as the stage 2 feature selection algorithm to balance the repeatability and false positive rate of the differential features. To test whether methylated genes and transcriptome profiling are significantly different between normal and tumor samples, we performed heatmap analysis on the differentially methylated genes of BRCA with 892 samples after the stage 2 feature selection (Figure 2A). We found that the level of the methylated gene in each normal or tumor samples was similar, and differentially methylated genes after stage 2 feature selection showed significant differences in normal and tumor tissues. Similarly, we implemented the volcano plot analysis on the transcriptome profiling of BRCA with 1,211 samples after the stage 2 feature selection (Figure 2B). Figure 2B indicates that the joint screening of |*log*_2_⁡*FC*| and *FDR* not only ensured the difference of features but also effectively controlled the overall false-positive rate.

#### Stage 3: mRMR

We adopted the mRMR algorithm (Peng et al., 2005) as the stage 3 feature selection, which could remove redundant features on the premise of preserving the regulation, modification, and collaborative associations in the triple dataset to the utmost extent. The mRMR algorithm maximizes the relevance between features *x_i_* and categorical target variables *t* while minimizing the redundancy between features by solving the equation as follows:

(4) $$max\left(\frac{1}{\left|S\right|}\sum_{x_{i}\in S}I\left(x_{i};t\right)-\frac{1}{{\left|S\right|}^{2}}\sum_{x_{i},x_{j\in S}}I\left(x_{i};x_{j}\right)\right)$$

where *I*(;;) is the mutual information of the two random variables, *S* is a feature subset, and |*S*| is the number of features in S. *I*(;) is defined in terms of the probabilistic density functions *p*(*x*),*p*(*y*), and *p*(*x*,*y*):

(5) $$\text{I}\left(\text{x};\text{y}\right)=\iint \text{p}\left(x,y\right)\text{log}\frac{\text{p}\left(x,y\right)}{\text{p}\left(\text{x}\right)\text{p}\left(\text{y}\right)}\text{dxdy}$$

The input data of the mRMR algorithm needed to be discrete. We defined the discrete function as follows:

(6) xi,j*={2   xi,j≥xj¯+k*δj,0       others,-2  xi,j≤xj¯-k*δj,

where δ_*j*_ represents the standard deviation of the *j*th feature, $\bar{x_{j}}$ is the average value of the *j*th feature, and *k* is the threshold parameter. In this study, we set *k* = 0.5, and the dataset has been discretized into three states{−2,0,2}.

### Heterogeneous Classifiers

After the hierarchical feature selection, we used four different models, i.e., SVM, decision tree (DT), random forest (RF), and AdaBoost, to capture the inherent information of the data with the characteristics of low dimension, nonlinear, imbalance class, and singular values. The large differences and obvious complementarity of the four models are as follows. First, SVM mapped data to higher dimensional space by kernel function and found the best separation hyperplane to maximize the margin between two types of training samples (Noble, 2006). SVM is good at solving the problems of low-dimension and nonlinear inseparable data. However, SVM is sensitive to the singular value, and its performance mainly depends on the selection of kernel function and related parameters. Second, DT was a tree structure, starting from the root node and recursively constructing from top to bottom. Non-leaf nodes were attribute features, with each branch representing the output of the judgment result, and leaf nodes were categories (Quinlan, 1986; Safavian and Landgrebe, 1991). DT was suitable for dealing with all kinds of discrete data. It has the characteristics of simple structure, strong interpretability, and few parameters. However, DT was biased toward the class with a large number of samples, was susceptible to singular values, and ignored the correlation between features (Breiman, 1996; Bauer and Kohavi, 1999; Dietterich, 2000b). Third, RF and AdaBoost were ensemble classifiers that used decision trees as individual classifiers. RF considered the correlation between features and had a high tolerance for singular values. However, if the training set of each tree was unbalanced in the process of random sampling, the performance would be very low on the small sample dataset (Breiman, 2001). Fourth, AdaBoost has certain adaptability for the unbalanced dataset, but it is sensitive to the abnormal samples.

### Second Learning Probability Error Ensemble Model

Existing ensemble strategies could not fully integrate the nonlinear relationships between different classifiers and unbiasedly estimate the nonlinear change rules of the triple dataset. Thus, we proposed the SLPEE. First, we combined the class probability prediction values of the first-learning heterogeneous classifiers and the actual class labels of the validating set to form a new dataset, so the diagnosis errors of individual classifiers were implicit in the new dataset. Then, we conducted a second-learning on the new data set *via* Xgboost, which used the residual between the real value and the predicted value as the next iteration of the learning goal. Xgboost could effectively learn errors and had a strong ability of nonlinear fitting, self-learning, and self-correction (Chen and Guestrin, 2016). Algorithm 1 described the SLPEE in detail. *D* was the data matrix, *y* was the label set that has two labels {−1,1}, corresponding to the normal and tumor samples, *h* was the heterogeneous classifier, and E represented the SLPEE model. *L_f_* was the learning set in the *f*th fold, Test_f_ was the testing set in the *f*th fold, and *T*_*fk*_ represented the *k*-fold training set in the *f*-fold learning set.

**Algorithm 1: The SLPEE based on F-fold CV.**

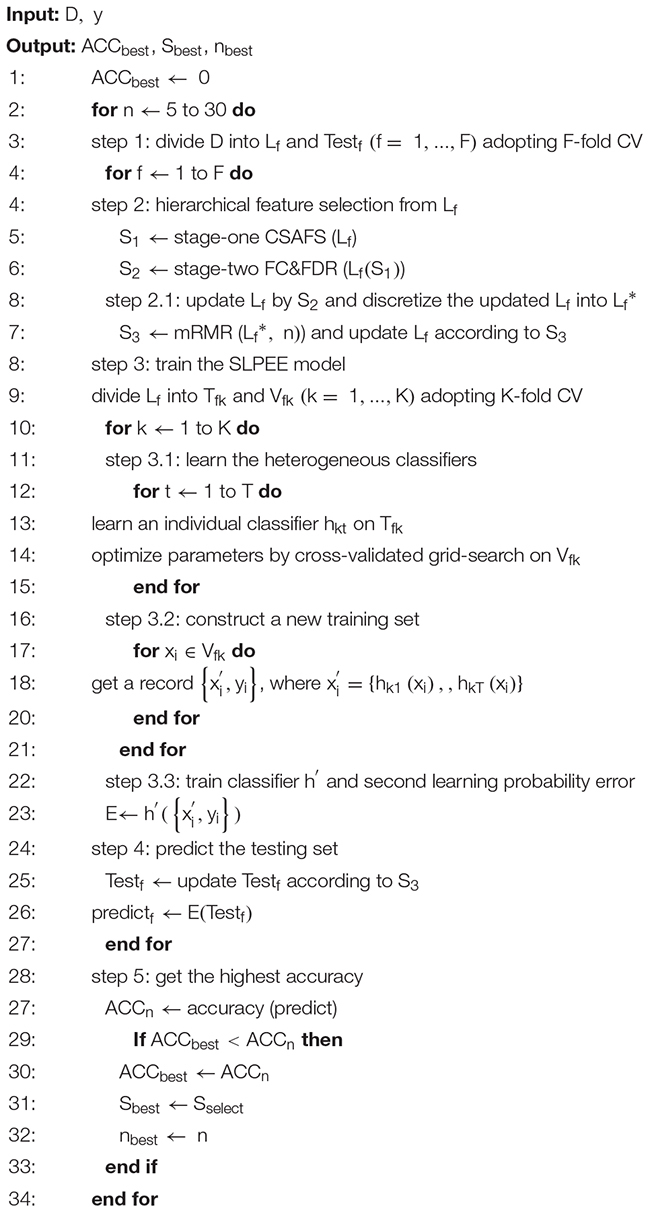

### Datasets and Preprocessing

We downloaded the protein-coding gene expression profiles, non-coding RNA expression profiles, and DNA methylation data of BRCA, LUAD, and KIRC from the TCGA official website^1^. For the transcriptome profiling, the amount of gene expression is HTSeq-Counts, and the total dimension of protein-coding gene and ncRNA expression profiles is 60,244. For the DNA methylation data, we used Illumina Human Methylation 450 array methylation chip data. The chip has 485,577 probes, which can detect nearly 450,000 methylation CpG sites in the entire human genome, covering 96% of CpG islands. Based on the same sample ID, we integrated the protein-coding gene expression profiles, non-coding RNA expression profiles, and DNA methylation data to construct a triple dataset. The sample numbers of the original datasets for the three cancers are shown in Table 1.

**TABLE 1:** Summary of the original different datasets for three cancers.

Datasets	No. of tumor	No. of normal	Total samples	Dimensions
		**BRCA**		
Protein-coding gene	1,109	113	1,222	19,676
ncRNA	1,109	113	1,222	40,568
DNA methylation	796	96	892	485,577
**The triple dataset**	**783**	**83**	**866**	**545,821**
		**LUAD**		
Protein-coding gene	535	59	594	19,676
ncRNA	535	59	594	40,568
DNA methylation	475	32	507	485,577
**The triple dataset**	**465**	**21**	**486**	**545,821**
		**KIRC**		
Protein-coding gene	539	72	611	19,676
ncRNA	539	72	611	40,568
DNA methylation	325	160	485	485,577
**The triple dataset**	**321**	**24**	**345**	**545,821**

*The triple dataset is the integrated data of the protein-coding gene expression profiles, non-coding RNA expression profiles, and DNA methylation. No. of tumor means the number of tumor samples. No. of normal means the number of normal samples. Total samples mean the total number of all the samples. Dimensions means the dimensions of every type of data.*

We preprocessed the samples and data successively. First, due to the limitations of experiments conditions and manual operation, some metastatic samples are wrongly classified as primary tumor samples, resulting in one patient who may correspond to multiple tumor samples. For example, in the DNA methylation dataset of BRCA, patient TCGA-BH-A1ES corresponds to two tumor samples, of which TCGA-BH-A1ES-06A-12D-A244-05 is a metastatic sample. In this work, we kept the primary tumor sample and the solid tissue normal sample related to the study and excluded outliers. Second, we preprocessed the data, including removing duplicate features, removing features with severe missing values, and correcting the normalized data. For example, when the missing value of a feature accounts for 100% of the total sample size, we think that the missing feature is too serious and should be deleted. We used the normalizeBetweenArrays function of the limma package in R to correct the normalized data. The datasets after preprocessing of the three cancers are shown in Table 2.

**TABLE 2:** Summary of preprocessed datasets for three cancers.

Datasets	No. of tumor	No. of normal	Total samples	Dimensions
		**BRCA**		
Protein-coding gene	1,098	113	1,211	19,676
ncRNA	1,098	113	1,211	40,568
DNA methylation	792	96	888	485,577
**The triple dataset**	**778**	**83**	**861**	**545,821**
		**LUAD**		
Protein-coding gene	517	59	576	19,676
ncRNA	517	59	576	40,568
DNA methylation	464	32	496	485,577
**The triple dataset**	**457**	**21**	**478**	**545,821**
		**KIRC**		
Protein-coding gene	531	72	603	19,676
ncRNA	531	72	603	40,568
DNA methylation	321	160	481	485,577
**The triple dataset**	**318**	**24**	**342**	**545,821**

*The triple dataset is the integrated data of the protein-coding gene expression profiles, non-coding RNA expression profiles, and DNA methylation. No. of tumor means the number of tumor samples. No. of normal means the number of normal samples. Total samples mean the total number of all the samples. Dimensions means the dimensions of every type of data.*

### Parameter Settings

In order to reproduce all experimental results in our paper, we set the specific random seeds for BRCA, KIRC, and LUAD as 14, 14, 20, and the other parameters setting of three cancers were same. In the stage 3 feature selection, we assumed that the size of the optimal feature subset is *n*, which increased from 5 to 30 with a step of one. Our proposed model optimizes the parameters of individual classifiers *via* grid-search over a parameter grid. For DT, we used the default parameters. For SVM, we set the kernel function as RBF, *C* = {0.001, 0.01, 0.1}, and *gamma* = {1.0, 10.0, 100.0}. For RF, we set n_estimators = {50, 100}. For Xgboost and AdaBoost, we, respectively, set n_estimators = {100, 200, 300} and n_estimators = 50.

### Performance Evaluation of HFS-SLPEE

#### Prediction of Cancer Diagnosis

In this work, we implemented 10-fold cross-validation (CV) on three cancers in TCGA to evaluate the prediction performance of HFS-SLPEE. In the 10-fold CV experiment, the triple dataset was randomly divided into 10-folds with equal size, 9 of which were taken as the learning set, and the remaining 1-fold was the testing set. The process is repeated 10 times until all samples are predicted once. In each learning set, we divided the learning set into *K* disjoint subsets by performing *K*-fold CV again, of which *K*−1 subsets were the training set and the remaining one subset was the validating set.

#### Evaluation Metrics

We considered that the primary tumor was a positive class, and the solid normal tissue was the negative class. There were four results of cancer diagnosis in the testing set: true positive (TP), true negative (TN), false negative (FN), and false positive (FP). Among the results, TP represented the number of correctly classified tumor tissue samples, TN denoted the number of correctly classified normal tissue samples, FN indicated the number of samples predicted to be normal tissue but actually tumor tissue, and FP stood for the number of samples predicted to be tumor tissue but actually normal tissue. Therefore, accuracy, sensitivity/recall, specificity, and F1-score were defined as follows:

(7) $$Accuracy=\frac{TP+TN}{TP+FP+TN+FN}$$

(8) $$Sensitivity/Recall=\frac{TP}{TP+FN}$$

(9) $$Specificity=\frac{TN}{TN+FP}$$

(10) $$F1-score=2\cdot \frac{Precison\cdot Recall}{Precision+Recall}=\frac{2TP}{2TP+FP+FN}$$

## Results

### Performance of HFS-SLPEE Based on the Cross-Validation

**FIGURE 3:**
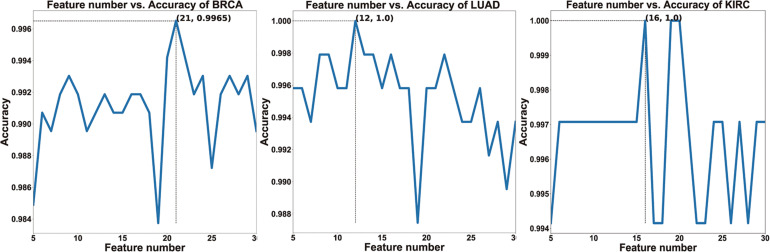
The relationship curves of the features and the accuracy of three cancers.

To verify whether HFS-SLPEE can generalize the diagnosis of different cancers, we researched on three high-incidence cancers BRCA, LUAD, and KIRC. We recorded the accuracy corresponding to the feature variable *n* (see Figure 3). It indicates that as n continues to increase, the accuracy increases; when *n* = 21, *n* = 12, *n* = 16, the accuracy reached the peak value and then show a downward trend, which means that the added features contain more noise than information. In this study, we take the features subset when the highest point is reached at first as the optimal. That is, *n* = 21, *n* = 12, *n* = 16, respectively, as the number of features finally selected for the BRCA, LUAD, and KIRC.

The predicted results of HFS-SLPEE for three cancers in TCGA are shown in Table 3. We used 21 key features to make BRCA achieve 99.65% accuracy, 99.61% sensitivity, 100% specificity, and 99.81% F1-score, and only three samples were misdiagnosed. For LUAD and KIRC, we selected 12 and 16 key features to achieve four-indicator 100% precision diagnosis. The results show that HFS-SLPEE achieves an excellent performance and has the generalization ability for three high-incidence cancers diagnosis.

**TABLE 3:** The diagnosis results of three cancer by HFS-SLPEE (%).

Metrics	BRCA	LUAD	KIRC
No. of features	21	12	16
$\left[\begin{array}{cc} TN & FP\\ FN & TP \end{array}\right]$	$\left[\begin{array}{cc} 83 & 0\\ 3 & 775 \end{array}\right]$	$\left[\begin{array}{cc} 21 & 0\\ 0 & 457 \end{array}\right]$	$\left[\begin{array}{cc} 24 & 0\\ 0 & 318 \end{array}\right]$
Accuracy	99.65%	100%	100%
Sensitivity	99.61%	100%	100%
Specificity	100%	100%	100%
F1-score	99.81%	100%	100%

### Performance of HFS-SLPEE by Ablation Analysis

Our proposed approach mainly consists of three parts, namely, the triple dataset (TDS), the novel hierarchical feature selection algorithm, and SLPEE model. To examine the contribution of each component, we compared the proposed approach with several combinations.

**FIGURE 4:**
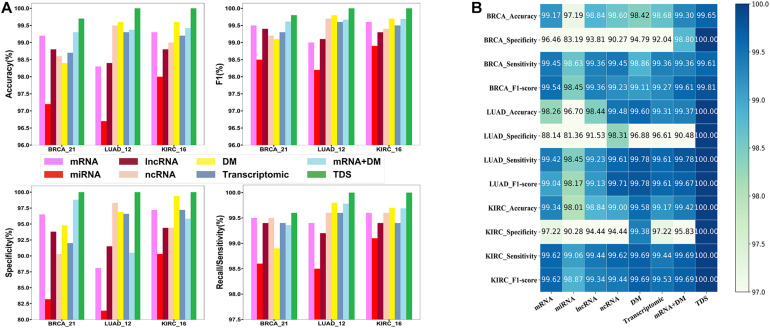
The comparison results of different datasets. **(A)** The histogram of comparison results. **(B)** The annotated heatmap of comparison results. As is shown, compared with the other seven datasets, the integrated data of the protein-coding gene expression profiles, non-coding RNA expression profiles, and DNA methylation can improve the performance of the model.

First, we compared the triple dataset with the other seven datasets, including mRNA, miRNA, lncRNA, ncRNA, DNA methylation, transcriptomic, and mRNA and DNA methylation, to inspect the contribution of the triple dataset. We found that the triple dataset achieved the best performance compared with the other seven datasets (see Figures 4A,B). Specifically, miRNA, mRNA, ncRNA, and DNA methylation are all single-type datasets with no absolute dominance, and their contribution rates in different cancers and diagnostic performances are different. The duplex-type datasets (transcriptomic, mRNA, and DNA methylation) have improved performance in many cases compared with the contained single-type dataset. The results indicated that the triple dataset contained more comprehensive and useful information and provided a robust data support.

Next, to examine the contribution of CSAFS, which saves the information of all methylation CpG sites related to genes in an aggregated form, we compared the hierarchical feature selection algorithm without and with CSAFS. Owing to the BRCA with the largest number of samples among three cancers, we take BRCA as an example, when the feature variable *n* is 21, to compare the performance of diagnosis and the time of the feature selection process. On the one hand, the experimental results showed that the hierarchical feature selection with CSAFS achieved better performance, and the accuracy, sensitivity, specificity, and F1-score of BRCA are improved by 0.23, 0.12, 1.2, and 0.13%, respectively. In the final feature subset selected by the hierarchical feature selection with CSAFS, we screened out 11 methylated genes such as WT1-AS and AL513523.2, accounting for about 30% of the final feature subset. On the other hand, the time consumed by the hierarchical feature selection without and with CSAFS is 2.6 and 0.15 h, respectively. It indicated that CSAFS, as the stage 1 feature selection approach, is capable of non-destructively preserving the complex and essential many-to-many modification associations between methylation CpG sites and genes and improving the efficiency of cancer diagnosis.

**FIGURE 5:**
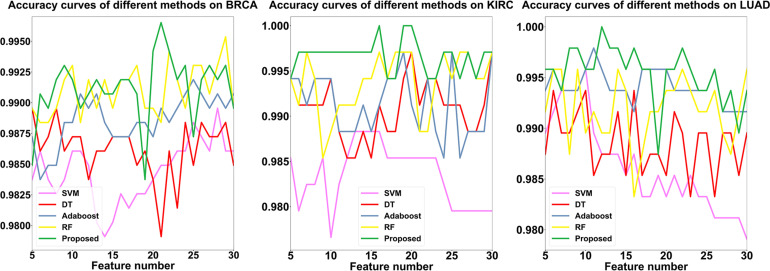
The comparison results of SLPEE and the other four models.

Finally, to verify the contribution of SLPEE further, we compared the accuracy of SLPEE with DT, RF, SVM, and Adaboost by using the triple dataset and the proposed hierarchical feature selection algorithm that traversed each feature in the range of 5–30. The results showed that the four models have different accuracies for different cancers with different feature numbers and have significant differences and complementary effects (see Figure 5). We found that the SLPEE accounts for 61.5, 88.5, and 92.3% with higher accuracy than the other four models for BRCA, KIRC, and LUAD datasets, respectively. That is, the accuracies of SLPEE model for three cancers are generally better than the other four models, which fully reveals that SLPEE absorbs the advantages of four models and overcomes their respective shortcomings. SLPEE breaks through the limitation of the single model with a limited scope of application and enhances the generalization of different features and different types of cancer diagnoses.

### Comparison With the Published State-of-the-Art Research

We compared the performance of HFS-SLPEE with state-of-the-art research (Raweh et al., 2018; Xiao et al., 2018b; Alghunaim and Al-Baity, 2019; see Table 4). Table 4 shows that most of the latest studies generally gave a single performance indicator and at most three performance indicators. In this study, we presented four performance indicators to comprehensively measure the performance of HFS. Alghunaim and Al-Baity (2019) used protein-coding gene expression profiles, DNA methylation, and their integrated data in TCGA and treated all data as features with SVM, DT, and RF three models for BRCA diagnosis. Comparing with Alghunaim and Al-Baity (2019), Table 4 shows that the accuracy and sensitivity of the HFS-SLPEE based on the protein-coding gene expression profiles and DNA methylation data are improved by 1.97 and 2.54%, respectively. The triple dataset was an effective solution to the shortcomings of the duplex dataset, which not only achieved the same 100% specificity but also improved the accuracy and sensitivity by 2.32 and 2.79%, respectively. Raweh et al. (2018) proposed a hybrid feature selection algorithm based on the DNA methylation data and applied naive base, RF, and SVM for BRCA, LUAD, and KIRC diagnosis. Comparing with Raweh et al. (2018), we found that HFS-SLPEE steadily improved the accuracy and F1-score by 0.03–0.35% and 0.29–4.21% for the three cancers, respectively. Xiao et al. (2018b) utilized the DESeq feature selection method to screen for the differentially expressed genes and adopted a deep neural network to learn the predictions of KNN, SVM, DT, RF, and GBDT. It was indicated that HFS-SLPEE improved the accuracy by 0.76%, comparing with the results of Xiao et al. (2018b). In summary, the proposed approach is a powerful framework for precision cancer diagnosis, which refers to the three aspects of the dataset, feature selection, and diagnosis model, outperforming the previously published state-of-the-art methodologies.

**TABLE 4:** Comparison results with the state-of-the-art approaches (%).

Cancer	Datasets	Methods	Accuracy	Sensitivity	Specificity	F1
BRCA	mRNA	Xiao et al., 2018b	98.41	–	–	–
	mRNA	Proposed	**99.17**	**99.45**	**96.46**	**99.54**
	DM	Raweh et al., 2018	98.33	–	–	94.90
	DM	Proposed	**98.42**	**98.86**	**94.79**	**99.11**
	mRNA + DM	Alghunaim and Al-Baity, 2019	97.33	96.82	**100**	–
	mRNA + DM	Proposed	**99.30**	**99.36**	98.80	**99.61**
	Transcriptome + DM	Proposed	**99.65**	**99.61**	**100**	**99.81**
LUAD	DM	Raweh et al., 2018	99.25	–	–	96.50
	DM	Proposed	**99.60**	**99.78**	**96.88**	**99.78**
	Transcriptome + DM	Proposed	**100**	**100**	**100**	**100**
KIRC	DM	Raweh et al., 2018	99.55	–	–	99.40
	DM	Proposed	**99.58**	**99.69**	**99.38**	**99.69**
	Transcriptome + DM	Proposed	**100**	**100**	**100**	**100**

*DM is the abbreviation of DNA methylation; The best performance in each case is given in boldface.*

### HFS-SLPEE Uncovers New Potential Biomarkers for Three Cancers

According to the optimal feature variable, we obtained 10 group features corresponding to the BRCA, LUAD, and KIRC with a total of 210, 120, and 160 features. After removing the overlapping features, the optimal features subsets were, respectively, reduced to 37, 35, and 40 features for the three cancers. The average repetition rate of the 10 group features for three cancers is 70.83–82.38%. On the one hand, the features selected in each fold is different, verifying that cancer does not generate along a fixed trajectory, but there are many different signal pathways. On the other hand, although the features subset selected in each fold changes dynamically, some feature genes recurring, and part of them will appear steadily in every fold.

We organized the features into three categories: protein-coding genes, ncRNAs, and methylated genes (see Table 5). We have two findings. First, the proportion of epigenetic factors containing DNA methylation and non-coding genes in carcinogenesis is not less than 1/3. Specifically, the proportion of epigenetic factors (including methylated genes and non-coding genes) in features subset are 40.54, 34.29, and 37.5% for BRCA, LUAD, and KIRC, respectively. Second, a limited number of specific protein-coding genes and lncRNAs (antisense and or lincRNA) appear steadily in each cross-validation experiment, which can be regarded as potential biomarkers for these three cancers.

**TABLE 5:** The summary of selected features for the three cancers.

Cancer	Protein-coding genes	ncRNAs	Methylated genes
BRCA	**MME, C1QTNF9, FIGF, SDPR, CHL1, MAMDC2, FMO2, PAMR1, ADAMTS5, TSLP, CD300LG, GLRA4**, PGM5P4(9), HPSE2(9), LYVE1(9), CLEC5A(3), WISP1(3), SCARA5(3), BTNL9(2), ADH1C(2), CILP2(1), HAGHL(1), CCL11(1), RP11-138I17.1(1)	**ADAMTS9-AS2| antisense, RP11-159H22.2| antisense**	WT1-AS (5), AL513523.2(5), F13A1(4), ABCB10P4(4), PABPC5(2), OR14I1(1), NID2(1), AC005796.2(1), AC079922.3(1), CCDC181(1), IGHV3OR16-10(1)
LUAD	**FAM107A, HBA1, CD5L, GPM6A**, ODAM(9), GRHL3(6), RAB26(5), ANKRD1(3), CTD-3214H19.16(3), HBM(3),SSTR4(3), ADRB1(2), MB(1), SERTM1(1), FPR2(1), SH3GL3(1), MYOC(1), UPK3B(1), TFAP2A(1), LYVE1(1), SLC4A1(1), SLC6A4(1), ALKAL2(1)	**CTB-43E15.1| lincRNA**, RP11-371A19.2| antisense(8), RP11-203H2.2| lincRNA(6), LL22NC03-104C7.1| antisense(2), GS1-600G8.5| lincRNA(2), FGF10-AS1| antisense(1), PACRG-AS3| antisense(1), RPL13AP17| transcribed_processed_pseudogene(1), RP11-416I2.1| lincRNA(1), RP11-35J10.7| sense_intronic(1), LINC00163| lincRNA(1)	RP11-344B5.3(1)
KIRC	**IRX2, PRR35, ACPP**, KNG1(9), SCNN1B(9), PIK3C2G(9), SLC9A4(9), KCNJ10(8), TFAP2B(7), AQP2(6), TMEM45B(5), TRPV6(4), AIF1L(2), SCNN1G(2), C9orf135(1), RASL11B(1), DUSP9(1), GABRA2(1), FAM46D(1), CASR(1), NELL1(1), CLDN8(1), ACOT12(1), RP11-536G4.1(1), FAM19A4(1)	**BMPR1B-AS1| lincRNA, RP11-527L4.6| lincRNA, RP11-469H8.6| antisense,** RP11-35J10.6| sense_intronic(6), RP1135J10.7| sense_intronic(2), AC103563.7| antisense(2), PTCSC3| lincRNA(1), LINC01020| lincRNA(1), IGKV3OR2-268| IG_V_gene(1), AC008991.1| lincRNA(1), LINC00864| lincRNA(1), CTD-2626G11.2| lincRNA(1), CTD-2007H18.1| lincRNA(1)	RP11-266E6.3(1), PIK3IP1-AS1(1)

*The bold font indicates the feature appears in each fold in the 10-fold cross-validation experiment. LYVE1 (9) represents that LYVE1 appears nine times in the 10-fold cross-validation.*

## Discussion

In this work, we developed a novel hierarchical feature selection and second learning probability error ensemble model, called HFS-SLPEE, for cancer diagnosis. HFS-SLPEE is a precision cancer diagnosis framework, constituted by the integrated data of protein-coding gene expression profiles, non-coding RNA expression profiles, and DNA methylation data, the novel HFS algorithm, and the SLPEE model. We experimentally studied three high-incidence cancer as BRCA, LUAD, and KIRC in the TCGA database. The results have demonstrated that HFS-SLPEE achieves higher performance in comparison to several state-of-the-art methodologies. Therefore, HFS-SLPEE could be a powerful tool for cancer diagnosis. Moreover, HFS-SLPEE is universal, not limited to the field of cancer diagnosis. It is also suitable for cancer subtype classification, tumor origin detection, etc.

Herein, we acknowledge some limitations of our proposed method. Since a recent related study (Liang et al., 2020) has demonstrated that sufficient samples may enhance performance of models. Despite the enormous availability of cancer datasets in TCGA, the number of samples is still not enough. As a machine-learning-based model, HFS-SLPEE needed much time to train because of the high number of combinations of trainable hyperparameters. The number of normal samples is much less than that of tumor samples in practice, which has been a big challenge when building a gold-standard dataset for cancer diagnosis. A study by Liang et al. (2017) showed that somatically acquired structural variation (SV) may induce tumor formation; we will explore some other data, such as copy number alteration (CNA) and SV, for better performances of HFS-SLPEE in future work.

## Data Availability Statement

Source data and code can be downloaded from https://github.com/luckymengmeng/HFS-SLPEE.

## Author Contributions

YM and MJ conceived the concept of the work. YM collected data, performed the experiments, and wrote the manuscript. MJ helped in revising and supervising the manuscript. Both authors contributed to the article and approved the submitted version.

## Conflict of Interest

The authors declare that the research was conducted in the absence of any commercial or financial relationships that could be construed as a potential conflict of interest.

## Footnotes

1 https://portal.gdc.cancer.gov/

## References

[B1] Alghunaim S. Al-Baity H. H. (2019). On the scalability of machine-learning algorithms for breast cancer prediction in big data context. *IEEE Access* 7 91535–91546. 10.1109/ACCESS.2019.2927080

[B2] Al-Juniad A. F. Qaid T. S. Al-Shamri M. Y. H. Ahmed M. H. Raweh A. A. (2018). Vertical and horizontal DNA differential methylation analysis for predicting breast cancer. *IEEE Access* 6 53533–53545. 10.1109/ACCESS.2018.2871027

[B3] Ang J. C. Mirzal A. Haron H. Hamed H. N. A. (2015). Supervised, unsupervised, and semi-supervised feature selection: a review on gene selection. *IEEE ACM Trans. Computat. Biol. Bioinform.* 13 971–989. 10.1109/TCBB.2015.2478454 26390495

[B4] Bauer E. Kohavi R. (1999). An empirical comparison of voting classification algorithms: bagging, boosting, and variants. *Mach. Learn.* 36 105–139. 10.1023/A:1007515423169

[B5] Baylin S. B. Ohm J. E. (2006). Epigenetic gene silencing in cancer–a mechanism for early oncogenic pathway addiction? *Nat. Rev. Cancer* 6 107–116. 10.1038/nrc1799 16491070

[B6] Ben-Dor A. Bruhn L. Friedman N. Nachman I. Schummer M. Yakhini Z. (2000). “Tissue classification with gene expression profiles,” in *Proceedings of the 4th Annual International Conference on Computational Molecular Biology*, (New York NY), 54–64. 10.1089/106652700750050943 11108479

[B7] Breiman L. (1996). Bagging predictors. *Mach. Learn.* 24 123–140. 10.1007/BF00058655

[B8] Breiman L. (2001). Random forests. *Mach. Learn.* 45 5–32. 10.1023/A:1010933404324

[B9] Cao Z. Wang Y. Sun Y. Du W. Liang Y. (2015). A novel filter feature selection method for paired microarray expression data analysis. *Int. J. Data Min. Bioinform.* 12 363–386. 10.1504/ijdmb.2015.070071 26510292

[B10] Chen T. Guestrin C. (2016). “Xgboost: a scalable tree boosting system,” in *Proceedings of the 22nd Acm Sigkdd International Conference on Knowledge Discovery and Data Mining*, (New York NY), 785–794. 10.1145/2939672.2939785

[B11] Cho S.-B. Won H.-H. (2003). “Machine learning in DNA microarray analysis for cancer classification,” in *Proceedings of the First Asia-Pacific Bioinformatics Conference on Bioinformatics 2003*, Vol. 19 (Darlinghurst, NSW), 189–198. 10.5555/820189.820213 26215079

[B12] Crick F. H. Barnett L. Brenner S. Watts-Tobin R. J. (1961). General nature of the genetic code for proteins. *Nature* 192 1227–1232. 13882203 10.1038/1921227a0

[B13] DeRisi J. Penland L. Bittner M. Meltzer P. Ray M. Chen Y. (1996). Use of a cDNA microarray to analyse gene expression. *Nat. genet* 14 457–460. 8944026 10.1038/ng1296-457

[B14] DeRisi J. L. Iyer V. R. Brown P. O. (1997). Exploring the metabolic and genetic control of gene expression on a genomic scale. *Science* 278 680–686. 10.1126/science.278.5338.680 9381177

[B15] Dietterich T. G. (2000a). “Ensemble methods in machine learning,” in *Proceedings of the International Workshop on Multiple Classifier Systems: Springer*, (Berlin: Springer), 1–15. 10.1007/3-540-45014-9_1

[B16] Dietterich T. G. (2000b). An experimental comparison of three methods for constructing ensembles of decision trees: bagging, boosting, and randomization. *Mach. Learn.* 40 139–157. 10.1023/A:1007607513941

[B17] Furey T. S. Cristianini N. Duffy N. Bednarski D. W. Schummer M. Haussler D. (2000). Support vector machine classification and validation of cancer tissue samples using microarray expression data. *Bioinformatics* 16 906–914. 10.1093/bioinformatics/16.10.906 11120680

[B18] Gao L. Ye M. Lu X. Huang D. (2017). Hybrid method based on information gain and support vector machine for gene selection in cancer classification. *Genomics Proteomics Bioinform.* 15 389–395. 10.1016/j.gpb.2017.08.002 29246519 PMC5828665

[B19] Huang M.-W. Chen C.-W. Lin W.-C. Ke S.-W. Tsai C.-F. (2017). SVM and SVM ensembles in breast cancer prediction. *PLoS One* 12:e0161501. 10.1371/journal.pone.0161501 28060807 PMC5217832

[B20] Lazar C. Taminau J. Meganck S. Steenhoff D. Coletta A. Molter C. (2012). A survey on filter techniques for feature selection in gene expression microarray analysis. *IEEE ACM Trans. Computat. Biol. Bioinform.* 9 1106–1119. 10.1109/TCBB.2012.33 22350210

[B21] Li L. Weinberg C. R. Darden T. A. Pedersen L. G. (2001). Gene selection for sample classification based on gene expression data: study of sensitivity to choice of parameters of the GA/KNN method. *Bioinformatics* 17 1131–1142. 10.1093/bioinformatics/17.12.1131 11751221

[B22] Liang Y. Qiu K. Liao B. Zhu W. Huang X. Li L. (2017). Seeksv: an accurate tool for somatic structural variation and virus integration detection. *Bioinformatics* 33 184–191. 10.1093/bioinformatics/btw591 27634948

[B23] Liang Y. Wang H. Yang J. Li X. Dai C. Shao P. (2020). A Deep learning framework to predict tumor tissue-of-origin based on copy number alteration. *Front. Bioeng. Biotechnol.* 8:701. 10.3389/fbioe.2020.00701 32850687 PMC7419421

[B24] Lu H. Chen J. Yan K. Jin Q. Xue Y. Gao Z. (2017). A hybrid feature selection algorithm for gene expression data classification. *Neurocomputing* 256 56–62. 10.1016/j.neucom.2016.07.080

[B25] Luo J. Huang W. Cao B. (2016). A novel approach to identify the miRNA-mRNA causal regulatory modules in cancer. *IEEE ACM Trans. Computat. Biol. Bioinform.* 15 309–315. 10.1109/TCBB.2016.2612199 28113985

[B26] Lyu H. Wan M. Han J. Liu R. Wang C. (2017). A filter feature selection method based on the maximal information coefficient and gram-schmidt orthogonalization for biomedical data mining. *Comput. Biol. Med.* 89 264–274. 10.1016/j.compbiomed.2017.08.021 28850898

[B27] McCarthy D. J. Smyth G. K. (2009). Testing significance relative to a fold-change threshold is a TREAT. *Bioinformatics* 25 765–771. 10.1093/bioinformatics/btp053 19176553 PMC2654802

[B28] Meng Y. Jin M. Tang X. Xu J. (2020). Degree-based similarity indexes for identifying potential miRNA-disease associations. *IEEE Access* 8 133170–133179. 10.1109/ACCESS.2020.3006998

[B29] Model F. Adorjan P. Olek A. Piepenbrock C. (2001). Feature selection for DNA methylation based cancer classification. *Bioinformatics* 17 S157–S164. 10.1093/bioinformatics/17.suppl_1.s157 11473005

[B30] Modelska A. Quattrone A. Re A. (2015). Molecular portraits: the evolution of the concept of transcriptome-based cancer signatures. *Brief. Bioinform.* 16 1000–1007. 10.1093/bib/bbv013 25832647 PMC4652618

[B31] Network C. G. A. (2012). Comprehensive molecular portraits of human breast tumours. *Nature* 490:61. 10.1038/nature11412 23000897 PMC3465532

[B32] Noble W. S. (2006). What is a support vector machine? *Nat. Biotechnol.* 24 1565–1567. 10.1038/nbt1206-1565 17160063

[B33] Norris A. W. Kahn C. R. (2006). Analysis of gene expression in pathophysiological states: balancing false discovery and false negative rates. *Proc. Natl. Acad. Sci.U.S.A.* 103 649–653. 10.1073/pnas.0510115103 16407153 PMC1334678

[B34] Peng H. Long F. Ding C. (2005). Feature selection based on mutual information criteria of max-dependency, max-relevance, and min-redundancy. *IEEE Trans. Pattern Anal. Mach. Intell.* 27 1226–1238. 10.1109/TPAMI.2005.159 16119262

[B35] Quinlan J. R. (1986). Induction of decision trees. *Mach. Learn.* 1 81–106. 10.1007/BF00116251

[B36] Raweh A. A. Nassef M. Badr A. (2018). A hybridized feature selection and extraction approach for enhancing cancer prediction based on DNA methylation. *IEEE Access* 6 15212–15223. 10.1109/ACCESS.2018.2812734

[B37] Safavian S. R. Landgrebe D. (1991). A survey of decision tree classifier methodology. *IEEE Trans. Syst. Man Cybern.* 21 660–674.

[B38] Saha I. Bhowmick S. S. Geraci F. Pellegrini M. Bhattacharjee D. Maulik U. (2015). “Analysis of next-generation sequencing data of miRNA for the prediction of breast cancer,” in *Proccedings of the International Conference on Swarm, Evolutionary, and Memetic Computing: Springer*, eds Panigrahi B. Suganthan P. Das S. Satapathy S. (Cham: Springer), 116–127. 10.1007/978-3-319-48959-9_11

[B39] Schena M. Shalon D. Heller R. Chai A. Brown P. O. Davis R. W. (1996). Parallel human genome analysis: microarray-based expression monitoring of 1000 genes. *Proc. Natl. Acad. Sci.U.S.A.* 93 10614–10619. 10.1073/pnas.93.20.10614 8855227 PMC38202

[B40] Stratton M. R. Campbell P. J. Futreal P. A. (2009). The cancer genome. *Nature* 458 719–724. 10.1038/nature07943 19360079 PMC2821689

[B41] Sun L. Zhang X. Qian Y. Xu J. Zhang S. (2019). Feature selection using neighborhood entropy-based uncertainty measures for gene expression data classification. *Inform. Sci.* 502 18–41.

[B42] Tang W. Wan S. Yang Z. Teschendorff A. E. Zou Q. (2018). Tumor origin detection with tissue-specific miRNA and DNA methylation markers. *Bioinformatics* 34 398–406. 10.1093/bioinformatics/btx622 29028927

[B43] Xiao Q. Luo J. Liang C. Li G. Cai J. Ding P. (2018a). Identifying lncRNA and mRNA co-expression modules from matched expression data in ovarian cancer. *IEEE ACM Trans. Computat. Biol. Bioinform.* 17 623–634. 10.1109/TCBB.2018.2864129 30106686

[B44] Xiao Y. Wu J. Lin Z. Zhao X. (2018b). A deep learning-based multi-model ensemble method for cancer prediction. *Comput. Methods Programs Biomed.* 153 1–9. 10.1016/j.cmpb.2017.09.005 29157442

[B45] Xu J. Cai L. Liao B. Zhu W. Wang P. Meng Y. (2019). Identifying potential MiRNA-disease associations with probability matrix factorization. *Front. Genet.* 10:1234. 10.3389/fgene.2019.01234 31921290 PMC6918542

[B46] Xu J. Wang Z. Li S. Chen J. Zhang J. Jiang C. (2018). Combinatorial epigenetic regulation of non-coding RNAs has profound effects on oncogenic pathways in breast cancer subtypes. *Brief. Bioinform.* 19 52–64. 10.1093/bib/bbw099 27742663

[B47] Xu J. Zhu W. Cai L. Liao B. Meng Y. Xiang J. (2020). LRMCMDA: predicting miRNA-disease association by integrating low-rank matrix completion with miRNA and disease similarity information. *IEEE Access* 8 80728–80738. 10.1109/ACCESS.2020.2990533

[B48] Yang P. Yang H. Y. Zhou B. B. Zomaya A. Y. (2010). A review of ensemble methods in bioinformatics. *Curr. Bioinform.* 5 296–308. 10.2174/157489310794072508

[B49] Yoon H.-J. Lim J. S. (2013). “Lymphoma cancer classification using NEWFM based filtering method,” in *Proceedings of the 2013 International Conference on Information Science and Applications (ICISA): IEEE)*, (Pattaya), 1–2. 10.1109/ICISA.2013.6579505

[B50] Zhang S. Wang J. Ghoshal T. Wilkins D. Mo Y.-Y. Chen Y. (2018). lncRNA gene signatures for prediction of breast cancer intrinsic subtypes and prognosis. *Genes* 9:65. 10.3390/genes9020065 29373522 PMC5852561

[B51] Zhao J. Cheng W. He X. Liu Y. Li J. Sun J. (2018). Construction of a specific SVM classifier and identification of molecular markers for lung adenocarcinoma based on lncRNA-miRNA-mRNA network. *Onco Targets Ther.* 11:3129. 10.2147/OTT.S151121 29872324 PMC5975616

[B52] Zhou M. Jin M. (2017). Holographic ensemble forecasting method for short-term power load. *IEEE Trans. Smart Grid* 10 425–434. 10.1109/TSG.2017.2743015

